# Epidemiology of Treated Diabetes Ocular Complications in France 2008–2018—The LANDSCAPE French Nationwide Study

**DOI:** 10.3390/pharmaceutics14112330

**Published:** 2022-10-28

**Authors:** Catherine Creuzot-Garcher, Pascale Massin, Mayer Srour, Florian Baudin, Corinne Dot, Sylvia Nghiem-Buffet, Jean-Francois Girmens, Cedric Collin, Anne Ponthieux, Cecile Delcourt

**Affiliations:** 1 Department of Ophthalmology, University Hospital, 21000 Dijon, France; 2 Cabinet d’Ophtalmologie de Breteuil, Centre Broca, Hôpital Lariboisière, 75013 Paris, France; 3 Department of Ophthalmology, Centre Hospitalier Intercommunal de Créteil, Université de Paris Est Créteil, 94000 Créteil, France; 4 Department of Ophthalmology, Desgenettes Military Hospital, 69003 Lyon, France; 5 Centre d’Imagerie et de Laser, 75015 Paris, France; 6 Department of Ophthalmology, INSERM-DGOS CIC 1423, Centre Hospitalier National d’Ophtalmologie (CHNO) des Quinze-Vingts, 75012 Paris, France; 7 IQVIA France, 92099 La Défense, France; 8 Novartis Pharma SAS, 8/10 rue Henri Sainte Claire Deville, 92563 Rueil-Malmaison, France; 9 Team LEHA, Bordeaux Population Health Research Center, UMR 1219, Institut National de la Santé et de la Recherche Médicale (Inserm), University of Bordeaux, 33000 Bordeaux, France

**Keywords:** diabetic macular edema, diabetic retinopathy, incidence, prevalence, France, drug consumption, nationwide study

## Abstract

Aim: LANDSCAPE aimed to estimate the annual incidence and prevalence of treated diabetic macular edema (DME) and proliferative diabetic retinopathy (PDR) between 2008 and 2018. Methods: This French nationwide observational study used data from the French National Health Insurance Databases covering 99% of the French population. Data about healthcare consumption were used to identify adults treated with anti-VEGFs or dexamethasone implants (for DME) and with pan-retinal photocoagulation (for PDR). All French patients newly treated between 2008 and 2018 were included. Incidence and prevalence of treated DME and PDR were estimated for the age-matched general population and the population with diabetes in France. Sociodemographic characteristics and medical history were described in both populations. Results: We identified 53,584 treated DME patients and 127,273 treated PDR patients between 2008 and 2018, and 11,901 DME and 11,996 PDR new incident patients in 2018. The treated DME incidence in 2018 was 2.5 per 10,000 in the general population and 37.3 per 10,000 in the population with diabetes. Prevalence in 2018 was 9.5 and 143.7 per 10,000 in the respective populations. Treated PDR incidence in 2018 was 2.3 per 10,000 in the general population and 31.2 per 10,000 in the population with diabetes. Prevalence in 2018 was 19.9 and 270.3 per 10,000 in the respective populations. Incidence and prevalence were not age-dependent. Incidence of treated PDR incidence was relatively stable from 2008–2018. Incidence of treated DME incidence rose from 2012–2018, probably due to widening access to newly available treatments, such as anti-VEGFs. Conclusions: We provide exhaustive nationwide data on the incidence and prevalence of treated diabetic ocular complications in France over a 10-year period.

## 1. Background

Ocular complications of diabetes were previously the leading cause of blindness in working-age adults. Cataracts, age-related macular degeneration (AMD) and glaucoma have since taken over as the principal causes of blindness in working-age adults [1]. Thanks to improved diabetes management, earlier detection of diabetic retinopathy and contemporary treatment options, such as vascular epithelial growth factor (VEGF) inhibitors, the proportion of adults aged 50 years and older with blindness and moderate-to-severe vision impairment due to DR has dropped throughout high-income countries such as France from 1990 to 2020. These gains, however, are threatened by the rising prevalence of diabetes, which is expected to increase by 45% between the years 2021 and 2045 worldwide, with a 13% increase expected in Europe [2].

The two principal forms of diabetic retinopathy that threaten vision are diabetic macular edema (DME) and severe diabetic retinopathy, including proliferative diabetic retinopathy (PDR). In France, the health authorities and Physician Societies set up programs at the end of the 1990s to screen and monitor ocular complications of diabetes. It is now recommended that patients with diabetes have annual ocular monitoring, including color fundus photography [3].

There are several treatment options for patients with DME, including intravitreal (IVT) inhibitors of vascular endothelial growth factor (anti-VEGF), such as ranibizumab (Lucentis^®^) and aflibercept (Eylea^®^), and IVT corticosteroid implants (dexamethasone (Ozurdex). In France, the health authorities fully reimburse prescriptions of IVT anti-VEGFs and corticosteroid implants for the treatment of DME, having reimbursed prescriptions for ranibizumab since 2012 and prescriptions for aflibercept and dexamethasone implants since 2015. There is no restriction on the number of reimbursed injections. The gold-standard treatment for PDR is pan-retinal photocoagulation, which is recommended in cases of severe NPDR where diabetic retinopathy can progress rapidly [3].

The prevalence and incidence of ocular complications of diabetes, such as PDR and DME, in France has been described in a few cohorts of people with diabetes [3,4,5,6]. However, these studies were monocentre or regional. Therefore, these studies were not exhaustive and could include selection bias, since the studies were conducted in expert centers for the treatment of diabetic ocular complications. Given the rising prevalence of diabetes and the risk of vision impairment from ocular complications, more detailed and exhaustive epidemiological information is needed to aid the planning of treatment strategies and decision making.

We aimed to provide exhaustive nationwide estimates of the incidence and prevalence of treated PDR and DME between 2008 and 2018, in both the general population in France and in the population of people with diabetes in France. To do this, we used health claims data from the national medico-administrative databases collecting all reimbursement claims in France.

## 2. Methods

### 2.1. Study Design and Data Sources

LANDSCAPE is a retrospective longitudinal population study which made use of the French National Health Insurance database (Système National des Données de Santé (SNDS)). The SNDS contains anonymized exhaustive individual data on all reimbursed health expenses for the approximately 99% of the French population covered by national health insurance, from birth or immigration until death or emigration [7].

Individual-level information contained in the SNDS includes demographic data (age, sex, date of birth, date of death, residence), outpatient data (reimbursed drug dispensations and numbers of units; attributions of long-term chronic disease; dates and natures of paramedical interventions and laboratory tests) and private and public hospitalization data (admission dates, durations, ICD-10 codes for main and associated diagnoses, medical acts), according to the International Classification of Diseases, revision 10 (ICD-10).

Data regarding the size of the general population (by age and sex) were sourced from the French National Institute of Statistics and Economic Studies (INSEE) [8]. Data on the size of the population with diabetes (including by age) were obtained from the French National Health Insurance database [9] and the French Public Health Agency (Santé Publique France) [10].

### 2.2. Identification of Treated DME and PDR Patients

We identified adults aged 18 years and over with treated DME and PDR covered by French national health insurance from the SNDS.

Individuals with diabetes (type 1 or type 2) were identified using a validated algorithm [11]. Individuals were defined as having treated DME if they had diabetes and at least one anti-VEGF treatment (ranibizumab or aflibercept) or dexamethasone implant (Ozurdex) reimbursed between 2012 and 2018. As no DME drugs were reimbursed prior to 2012, treated DME patients could only be identified from 2012 onwards. Individuals were excluded if they were aged ≥80 years at the first treatment (to exclude nAMD patients), if they had retinal diseases other than DME (ICD-10 codes H30-H36, except PDR) or if they received treatments for other macular diseases (dynamic phototherapy with verteporfin).

Individuals were defined as having treated PDR if they had diabetes and at least one treatment by pan-retinal photocoagulation.

Individuals were excluded if any of the following applied to them: high myopia (reimbursement for high-correction refractive glasses in recent years), non-infectious uveitis (ICD-10 code H30.0 or H30.1 or treatments for non-infectious uveitis (topical or systemic corticoids for at least 1 month) in the year of dexamethasone implant administration) or residency in the French overseas region Mayotte (due to incomplete SNDS data for residents of this region).

Patients were followed until the end of healthcare consumption (regardless of treatment), or until the patient died or emigrated, thus exiting the SNDS database.

### 2.3. Ethics and Data Protection

The study LANDSCAPE received approval from the French data protection agency (CNIL) and the French Institute of Health Data (INDS). Informed consent was not required for access to anonymized data in the SNDS.

### 2.4. Statistical Analyses

#### 2.4.1. Annual Incidence and Prevalence of Treated DME and PDR

Incidence was calculated as the number of newly treated DME and PDR patients over a calendar year per 10,000 people in the French general population aged ≥18 years in that year. Incidence was also expressed per 10,000 people in the French population with diabetes aged ≥18 years in that year.

Treated DME and PDR incidence per 10,000 people in the French general population were also analyzed by age (5-year age groups and cumulative age group), region in France, by classification of the patients’ residential areas and by the number of general practitioners and ophthalmologists in patients’ home areas.

Prevalence was expressed as the number of treated DME and PDR patients in the calendar year 2018 per 10,000 people in the French general population aged ≥18 years in 2018 and per 10,000 people in the French population with diabetes in 2018.

#### 2.4.2. Patient Characteristics and Comorbidities

Descriptive summaries of patient characteristics (age, sex, comorbidities) were produced. Comorbidities were described for incident patients in 2018. Comorbidities were identified via ICD-10 codes associated with hospitalizations or long-term disease status, or by reimbursed treatments.

#### 2.4.3. Statistics

Quantitative variables were represented as means, standard deviations (SDs), medians, quartiles and minima/maxima. Categorical variables were represented as counts and percentages. Analyses were performed using SAS Enterprise Guide^®^ version 7.1.

## 3. Results

### 3.1. Patient Characteristics

We identified 53,584 treated DME patients and 127,273 treated PDR patients between 2008 and 2018. By the end of 2018, 91.5% of treated DME patients and 78.8% of treated PDR patients were still being followed in this study, 4.1% DME and 8.8% PDR patients had died, and 4.4% DME and 12.4% PDR patients had no recorded healthcare consumption in the previous 12 months. The adult French population identified in 2018 as living with diabetes (both type 1 and type 2) numbered 3,192,475.

Of the annual incident patients in 2018, 57.8% and 59.0% of treated DME and PDR patients were male (Table 1). Mean (SD) incident age was 66.3 (9.6) years and 65.1 (13.0) years for treated DME and PDR patients, respectively.

In 2018, cardiovascular and renal comorbidities for treated DME and PDR patients were as expected for patients with diabetes, and ocular diseases were as expected for individuals in their sixties (Table 1). Hypertension was the most frequent comorbidity and was present in three quarters of patients. DME and PDR patients had the same ocular and systemic comorbidities.

### 3.2. Treated DME and PDR Incidence 2008–2018

Incident treated DME was identified in 11,901 individuals in 2018. Annual incidence was 2.5 per 10,000 (95% CI: 2.5–2.5) for the general population and 37.3 per 10,000 (95% CI: 37.3–37.3) for the population with diabetes (Table 2).

Incident treated PDR was identified in 11,996 individuals in 2018. Annual incidence was 2.3 per 10,000 (95% CI: 2.3–2.3) for the general population and 31.2 per 10,000 (95% CI: 31.2–31.2) for the population with diabetes.

Adjusting incidence to the age- and sex-distribution of the French population in 2018 did not notably change these results for treated DME or PDR.

**Figure 1:**
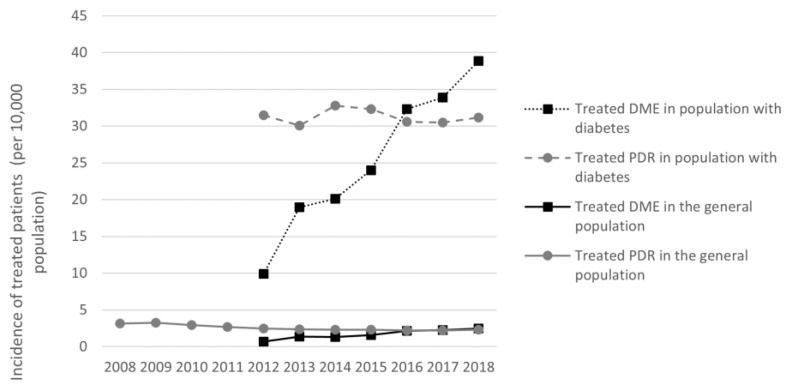
Treated DME and PDR incidence from 2008 to 2018 in the general population and in people with diabetes. Abbreviations: DME = diabetic macular edema, PDR = proliferative diabetic retinopathy. Incidence estimated for the corresponding general population in France and for the diabetic population aged ≥18 years.

In both the general population and the population with diabetes, treated PDR annual incidence was relatively stable between 2008 and 2018, while treated DME incidence increased from 2012 to 2018 (Figure 1).

**Figure 2:**
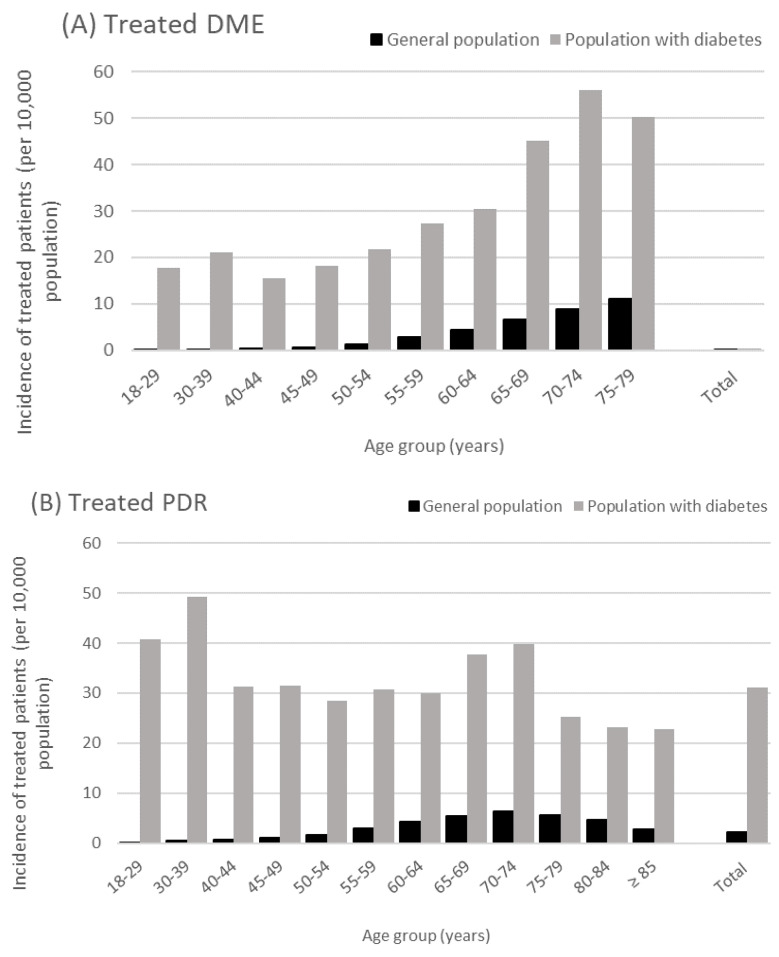
Treated DME (**A**) and PDR (**B**) incidence in 2018 per age category in the French general population and in the diabetic population. Abbreviations: DME = diabetic macular edema, PDR = proliferative diabetic retinopathy.

Treated DME incidence increased steadily with age. In 2018, treated DME incidence in people aged 70–74 years versus people aged 18–29 years was 90-fold higher in the general population and 2.8-fold higher in the population with diabetes (Figure 2). Similarly, treated DME incidence increased with cumulative age group. In 2018, treated DME incidence was 3.2-fold higher in people aged ≥70 years versus those aged ≥30 years in the general population, and was 1.4-fold higher in the population with diabetes (Table 2).

Treated PDR incidence in the general population in 2018 was maximal in people aged 70–74 years (6.3 per 10,000) and did not appear to be age-dependent among people with diabetes, although two peaks in incidence were observed around the ages 30–39 years and 65–74 years. Similarly, treated PDR incidence rose with cumulative age up to ≥70 years and then decreased, in both the general population and in the population with diabetes (Table 2).

### 3.3. Treated DME and PDR Prevalence in 2018

In 2018, we identified 45,868 individuals with treated DME and 103,980 individuals with treated PDR. Treated DME prevalence was therefore estimated at 9.5 per 10,000 in the general population and 143.7 per 10,000 in the population with diabetes. Treated PDR prevalence was estimated at 19.9 per 10,000 in the general population and 270.3 per 10,000 in the population with diabetes (Table 2).

Treated DME and PDR prevalence was highest for patients aged ≥70 years. In this age group, treated DME prevalence was 40.5 per 10,000 in the general population and 219.0 per 10,000 in the population with diabetes, and treated PDR prevalence was 54.7 per 10,000 and 313.7 per 10,000 in the respective populations.

### 3.4. Patients with Both Treated DME and PDR

In 2018, of the 45,868 treated DME and 103,980 treated PDR prevalent patients still followed in the SNDS, 16,123 had both treated DME and PDR, representing 35.2% of prevalent treated DME patients and 15.5% of prevalent treated PDR patients. For 54.9% of the treated DME + PDR patients, PDR appeared and was treated first, prior to DME, with 14.7 (15.9) months between first PDR treatment and first DME treatment. For patients with DME treated first, prior to PDR, DME treatment was initiated 9.7 (13.2) months prior to PDR treatment beginning.

### 3.5. Impact of Geography and Access to Medical Care

**Figure 3:**
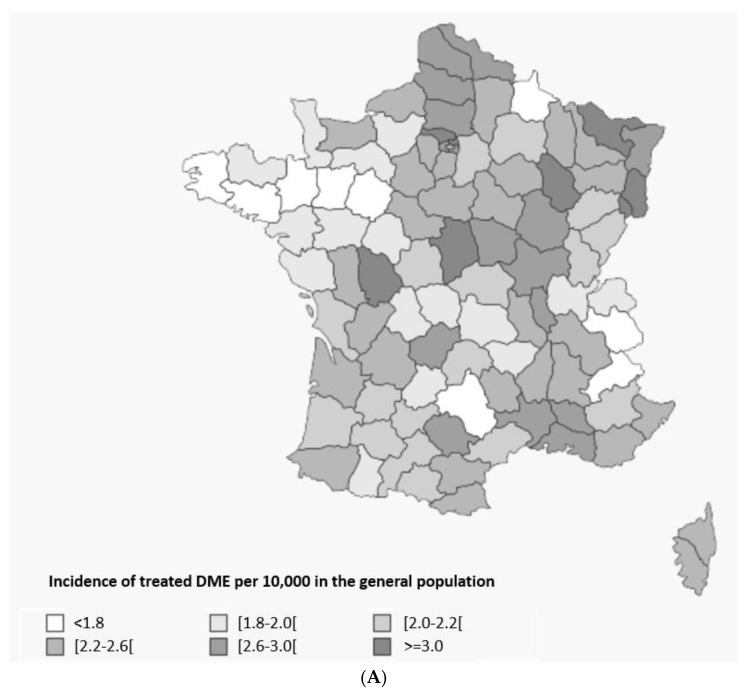

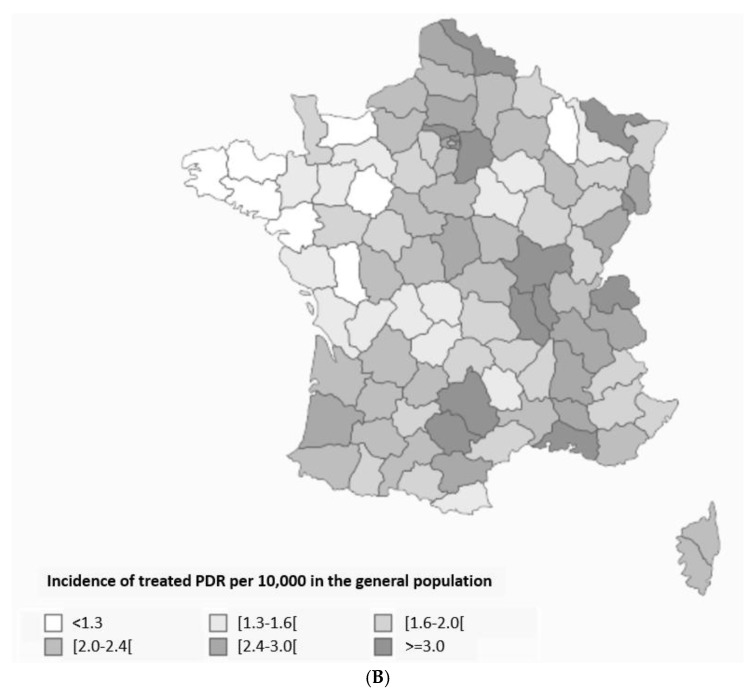
Treated DME (**A**) and PDR (**B**) incidence in the general population in 2018 per region in France. Abbreviations: DME = diabetic macular edema, PDR = proliferative diabetic retinopathy. Incidence in the corresponding population in France aged ≥18 years, expressed per 100 inhabitants.

Geographical trends were observed when treated DME and PDR incidence and prevalence were analyzed by French region. The incidences of both treated diseases were higher in Eastern France and the tip of Northern France and lower in Western France (Figure 3). Incidence of treated DME was 1.4-fold higher in the French overseas regions versus the whole of France (3.5 vs. 2.5 per 10,000), with a similar pattern observed for treated PDR (1.3-folder higher, 3.1 vs. 2.3 per 10,000).

**Figure 4:**
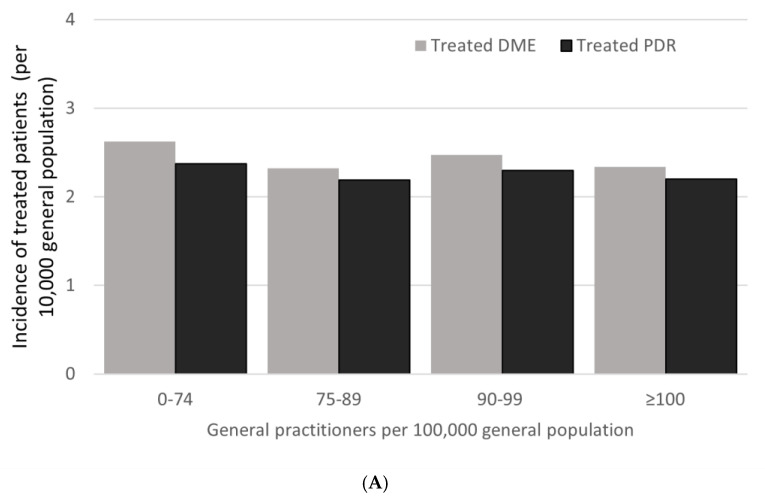

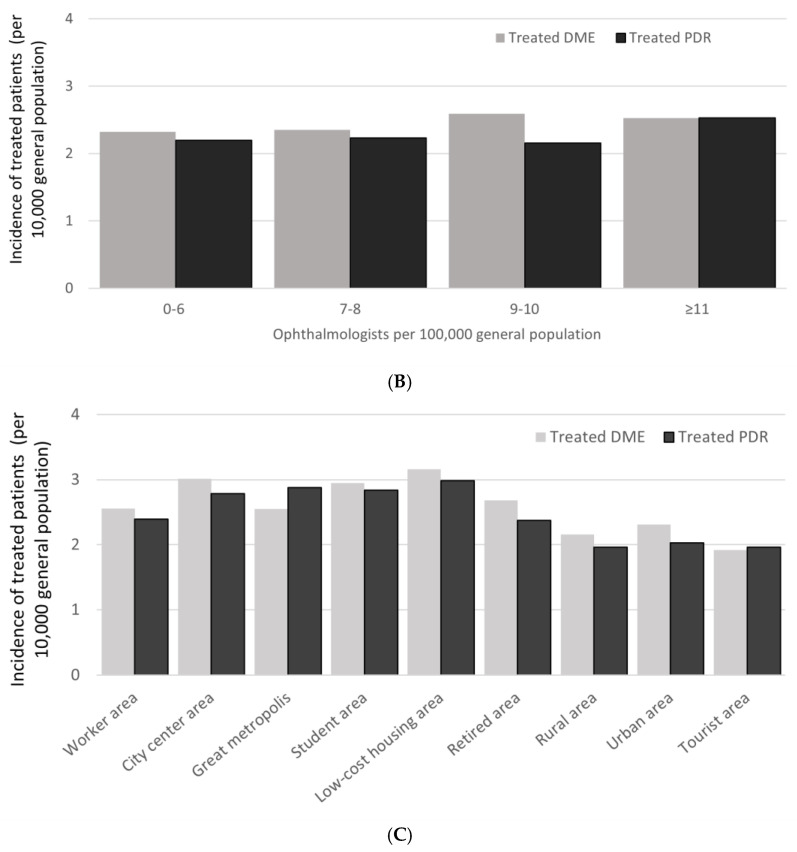
Treated DME and PDR incidence in the general population in 2018, by frequency of (**A**) general practitioners (GPs) and (**B**) ophthalmologists in residential area and (**C**) by type of patient residence.

Treated DME and PDR incidence did not appear to depend on the classification of the patient’s type of residence, nor on the density of general practitioners or ophthalmologists in the patient’s area (Figure 4).

## 4. Discussion

We used exhaustive, population-level reimbursement data from the French SNDS healthcare claims database to estimate the incidence and prevalence of treated DME between 2012 and 2018 and of treated PDR between 2008 and 2018. Treated DME incidence in 2018 was 2.5 per 10,000 people in the general population and 37.3 per 10,000 in the population with diabetes. Prevalence was 9.5 and 143.7 per 10,000 in the respective populations. Treated PDR incidence in 2018 was 2.3 per 10,000 in the general population and 31.2 per 10,000 in the population with diabetes. Prevalence in 2018 was 19.9 per 10,000 and 270.3 per 10,000 in the respective populations. The incidence of treated DME incidence rose from 2012 to 2018, whereas the incidence of treated PDR incidence was relatively stable from 2008 to 2018. Previous studies on DME and PDR have most often been conducted on small samples of patients treated from specific ophthalmological practices that may not be fully representative of patient management across the country. The present study also served to evaluate patterns associated with all approved DME treatments that are registered in the SNDS database. To our knowledge, DME and PDR epidemiology in the general population has not been previously reported; other studies have reported incidences in populations with diabetes. LANDSCAPE is therefore the first study to evaluate the incidence and prevalence of treated diabetic ocular complications in the general population as well as in the population with diabetes. Individuals identified with treated DME and PDR in LANDSCAPE had similar characteristics to the population with diabetes in France [9] in terms of gender distribution and comorbidities. The mean age of people in LANDSCAPE was similar to the mean ages observed in other, real-world French studies on DME [12,13,14,15,16].

The treated DME patients in LANDSCAPE correspond to populations in the literature described as having DME that is clinically significant, sight-threatening or requiring treatment. Furthermore, our analysis of incidence standardized by age group allowed direct comparison of LANDSCAPE results with previous studies.

### 4.1. Patient Identification and Characteristics

We identified treated DME and PDR patients by applying a detailed set of inclusion and exclusion criteria based on SNDS data on diagnosis and healthcare consumption. However, treated DME may have been missed or misidentified for some patients. For example, some diabetic patients may have been treated with a DME treatment for another condition, such as neovascular AMD or macular edema linked to vein occlusion. We minimized this risk using subsequent exclusion criteria based on age and other diagnoses (high myopia, AMD, treatment of other ocular disease, such as non-infectious uveitis). We may also have missed individuals with advanced DME diagnosed too late to be treated, or macular edema not significantly impacting visual acuity (DME IVTs are reimbursed in France for patients with visual acuity ≤70 ETDRS letters) and not treated by anti-VEGF or dexamethasone injections. In order to distinguish nAMD patients from DME patients when the only treatment was anti-VEGF, we excluded all patients initiating anti-VEGF treatment over 80 years from our DME population, as the mean age at initiation of anti-VEGF treatment was 80.1 years for nAMD patients [17] compared to 66.1 years for DME patients [12], with only 2% of DME patients initiating anti-VEGF after 80 years of age [12].

Identifying treated PDR patients was simpler and was based on treatment with at least one session of pan-retinal photocoagulation in individuals with diabetes. This strategy may have over-estimated pan-retinal photocoagulation incidence, since some patients with diabetes and severe non-proliferative diabetic retinopathy could also be treated with PRP to avoid neovascular conversion of retinopathy [18]. In addition, like DME, diabetic patients presenting with a vein occlusion can benefit from laser treatment in case of ischemic conversion.

### 4.2. Treated DME and PDR Epidemiology in People with Diabetes

Treated DME incidence in LANDSCAPE was 37.3 per 10,000 in the French adult population with diabetes in 2018. We observed an increase in treated DME incidence between 2012 and 2018. This is probably related to EURETINA guidelines to treat DME earlier [19], since delaying treatment can lead to deterioration of vision. During this period, access to ranibizumab became widespread, and aflibercept and dexamethasone implants became available and increasingly widespread from 2015. Therefore, we believe that the increased incidence of treated DME observed in LANDSCAPE corresponds to this widening access to effective DME therapies rather than to a rise in the incidence of DME itself. The focus on treated DME also explains why the 2018 incidence reported in LANDSCAPE (37.3 per 10,000 in the diabetic population) is lower than estimates from other studies, which have reported incidences ranging from 0.8% in the UK [20] to 1.8% in US Latinos aged over 40 years [21] and 2.7% and 2.2% in Spain among type 1 and type 2 diabetic patients, respectively [22]. In contrast, the incidence of treated DME in LANDSCAPE was similar to the pooled annual incidence of 0.4% from the Li et al. meta-analysis based on four studies with incidence data [23].

LANDSCAPE also showed that treated DME incidence increased with age, peaking at 70–74 years. This is probably due to the duration of diabetes, although, unfortunately, we were unable to measure diabetes duration in our study.

We estimated the 2018 prevalence of treated DME to be 143.7 per 10,000 in the population with diabetes. This sits in the mid-range of prevalence estimates reported elsewhere. Again, this could be partly because LANDSCAPE reported prevalence for treated DME, whereas other studies have reported prevalence for diagnosed DME. The variation in the literature could also be due to different diagnostic methods. Most studies used color retinography to diagnose DME, whereas the most recent studies used OCT. There are also differences between the study populations, in terms of duration of diabetes and HbA1c levels. Prevalence was 1.2% in a global meta-analysis based on retinal color photography [24] and 3.7% in a recent European meta-analysis [23], although the pooled estimate for French adults aged ≥40 years was 1.3%, closer to the LANDSCAPE estimate. Li et al. noted that prevalence of DME was lower in France compared to other large European countries and hypothesized that prevalence may be underestimated due to the lack of a nationwide screening program [23]. Another global meta-analysis estimated DME prevalence at 5.46% after the year 2000 [25]. Studies conducted after the year 2000 on color fundus photography yielded DME prevalences in people with diabetes ranging from <1% [26,27,28] and 1–2% [29,30] to 4–5% [31,32].

### 4.3. Treated PDR Incidence and Prevalence

In LANDSCAPE, the treated PDR incidence in 2018 was 31.2 per 10,000 in the French adult population with diabetes. This is in agreement with other studies which estimated PDR (or equivalent) incidence in people with diabetes at ≤0.5% [23,27,33]. A higher incidence was reported by Romero et al. for people with type 1 and type 2 diabetes (5.77% and 2.64%, respectively) [22]. A Paris-based screening program for diabetic retinopathy, in operation since 2004, estimated the 10-year annual incidence of referable DR (including earlier non-proliferative forms of DR as well as PDR) at 3.92% [5].

In LANDSCAPE, the incidence of treated PDR did not appear to be age-dependent. However, we observed two peaks in incidence. A peak in the fourth decade of life could correspond to diabetic retinopathy developing in people who have lived with type 1 diabetes since childhood. The other peak in the seventh decade of life could correspond to diabetic retinopathy developing in people who developed type 2 diabetes in their fifties. Numerous articles show that PDR is related to the duration of diabetes rather than to age [20,22,26,34,35].

In LANDSCAPE, the incidence of treated PDR appeared to be stable from 2008 to 2018, supporting previous reports of stable incidence over the decade from 2004 to 2014 in the UK [36]. The stable incidence of treated PDR also reflects the stable treatment regimen preceding and during the treatment period, with no major changes in management or treatment during this time.

The LANDSCAPE-reported treated PDR prevalence was 270.3 per 10,000 in the French adult population with diabetes. Again, this sits mid-way between previously reported prevalence estimates in people with diabetes. Global meta-analyses yielded prevalences of 1.7% [24] and 6.69 [25], while a European meta-analysis yielded a prevalence of 2.2% [23]. National studies of electronic healthcare databases estimated PDR prevalence at 1% in Denmark [29] and 8.1% in the UK [36]. Epidemiological studies based on images (mostly color fundus photography) estimated prevalence from 1% [26,32] up to 3% [30,31,35,37], depending on the type and duration of diabetes. Limited data are available for PDR prevalence in France; the only available French data are from a monocentre study, with an estimated prevalence of PDR at 17.6% in hospitalized people with type 1 diabetes [38].

### 4.4. Treated DME and PDR Epidemiology in the General Population

In LANDSCAPE, the prevalence of treated PDR was 19.9 per 10,000 in the adult general population of France. To our knowledge, only one previous study has estimated treated PDR prevalence in the general population. This UK study, using an electronic health database of primary care records, estimated the prevalence of severe diabetic retinopathy to be 0.1% in the general population [36].

### 4.5. Impact of Geography and Access to Care

In LANDSCAPE, the incidence of treated DME and PDR in 2018 was higher in Eastern and Northern France. This probably reflects the geographic distribution of diabetes, which is highest in the North and East of France, as well as in disadvantaged areas in big cities (north-eastern Paris and Marseille) [39]. When incidence was analyzed according to the socio-economic classification of the patients’ type of residence, however, incidence did not vary markedly. Treated DME and PDR were also more prevalent in the French overseas regions compared to the whole of France. Again, this could be due to high levels of diabetes in these regions [39], as well as possible contributions from ethnic variability and genetic and environmental factors, such as obesity and access to healthcare.

On a wider scale, the European meta-analysis by Li et al. showed that DME and PDR prevalence was lower in France compared to other large European countries, possibly due to the lack of a nationwide screening program [23]. Indeed, the ophthalmological follow-up of people with diabetes is insufficient in France, with <50% of patients having an annual examination and <66% having a biennial examination [39]. A global meta-analysis showed that Europe and South-East Asia had the lowest rates of any diabetic retinopathy worldwide [24].

Proximity to general or ophthalmic medical care did not affect treated DME or PDR incidence in France in 2018. This suggest that patients can access care and treatment as required. Given the predicted growth in diabetes prevalence, however, healthcare investment may be required to maintain current standards of access to care in France.

## 5. Strengths and Limitations

The SNDS medico-administrative database, where all health care resources are registered for reimbursement purposes for the entire French population, is a powerful epidemiological tool and is being increasingly used to describe and follow up ophthalmic conditions and cares, especially IVT in retinal diseases [3,40] and in diabetes [41]. The SNDS avoids selection bias, which can affect observational cohort studies, allowing huge, robust, longitudinal cohorts with minimal attrition and providing granular data on gender, age, geographical location and access to medical care.

This study has several limitations. First, this study did not aim to describe all DME and PDR patients but only those treated with IVTs and PRP, as disease diagnosis information was not directly available in the databases; thus, LANDSCAPE probably underestimates DME and PDR incidence and prevalence, particularly compared to studies using systematic eye examinations and imaging. This is probably offset by the fact that nearly all patients in France diagnosed with DME and PDR are treated, since the French system of fully reimbursed healthcare removes any economic barriers to accessing treatment.

## 6. Conclusions

In conclusion, this comprehensive study, conducted at the scale of the entire French population using drug consumption data, reflects treated incidence and prevalence of the diabetic ocular complications DME and PDR in 2018 and over the decade 2008–2018. Our data suggest that treated PDR incidence is stable in France, whereas treated DME incidence has increased since the introduction of new therapies and guidelines to treat DME earlier. As the global prevalence of diabetes is predicted to continue to increase in forthcoming decades, recent exhaustive epidemiological data, such as the results from the LANDSCAPE French nationwide study, will be crucial for planning healthcare delivery to prevent vision loss due to DME and PDR.

## Figures and Tables

**Table 1:** Demographics and characteristics of incident treated DME and PDR patients in 2018.

		Treated DME Patients N = 11,901	Treated PDR Patients N = 11,996
Sex	Male	6881 (57.8%)	7078 (59.0%)
	Female	5020 (42.2%)	4918 (41.0%)
Age, years	N	11,901 (100.0)	11,996 (100%)
	Mean (SD)	66.3 (9.6)	65.1 (13.0)
	Median (Q1–Q3)	68.0 (61.0–73.0)	66.0 (58.0–74.0)
	Range	(18.0–79.0)	(18.0–99.0)
Ocular diseases	Cataract surgery ^1^	3401 (28.6%)	3284 (27.4%)
	Treated dry eye disease ^2^	3608 (30.3%)	2683 (22.4%)
	Treated ocular hypertension ^2,3^	1845 (15.5%)	1835 (15.3%)
Non-ocular diseases	Hypertension ^4^	9329 (78.4%)	9065 (75.6%)
	Acute coronary syndrome	509 (4.3%)	567 (4.7%)
	Chronic coronary syndrome	1874 (15.7%)	1910 (15.9%)
	Obliterating peripheral arterial disease	845 (7.1%)	930 (7.8%)
	Stroke ^5^	1476 (12.4%)	1821 (15.2%)
	Dementia	160 (1.3%)	224 (1.9%)
	Renal disease	1535 (12.9%)	1637 (13.6%)
	Non-metastatic cancer	1533 (12.9%)	1471 (12.3%)

Abbreviations: DME = diabetic macular edema, PDR = proliferative diabetic retinopathy, Q = quartile, SD = standard deviation. ^1^ Cataract surgery reported from 2008 to 2018. ^2^ Treated in 2018. ^3^ Including glaucoma. ^4^ Patients treated with antihypertensives. ^5^ Including transient ischemic stroke.

**Table 2:** Treated DME and PDR incidence and prevalence in 2018 by age group. Incidence and prevalence of treated DME and PDR in 2018 are presented by cumulative age group and by age group. Incidence and prevalence are presented per 10,000 adults in the general population in France and per 10,000 adults with diabetes in France.

	Treated DME Annual Incidence		Treated DME Prevalence		Treated PDR Annual Incidence		Treated PDR Prevalence	
	Patients Treated for Condition per 10,000 Population							
	General Population	Population with Diabetes	General Population	Population with Diabetes	General Population	Population with Diabetes	General Population	Population with Diabetes
**Cumulative age groups (years)**								
Total ≥ 18 (All)	2.5	37.3	9.5	143.7	2.3	31.2	19.9	270.3
≥30	3	37.5	11.7	145	2.8	31.1	24.1	271.6
≥40	3.8	38	14.7	146.9	3.3	30.7	29	270.1
≥50	5.1	39.8	19.9	155.1	4.1	30.6	36.8	275.2
≥60	7.2	44.7	28.6	177.7	5	30.9	47.6	295.6
≥70	9.8	53.2	40.5	219	5	28.7	54.7	313.7
≥80	-	-	-	-	3.7	23	46.8	289.8
**Age groups (years)**								
18–29	0.1	17.7	0.2	44.1	0.2	40.8	0.7	153.7
30–39	0.2	21	0.7	71.8	0.5	49.3	3.3	342.1
40–44	0.3	15.5	1.1	49.8	0.7	31.3	4.6	217.9
45–49	0.6	18.1	2.1	58.2	1.1	31.6	6.8	189.5
50–54	1.3	21.7	4.6	75.1	1.7	28.4	11.3	185
55–59	2.7	27.4	9.3	94	3	30.7	19.8	200.3
60–64	4.4	30.4	16.6	114.5	4.3	30	32	221.4
65–69	6.6	45.1	25.3	173.4	5.5	37.8	46.9	320.6
70–74	9	56.1	36.8	232.2	6.4	39.8	60.8	383.2
75–79	11.8	50.3	45.6	205.7	5.7	25.2	61.2	276.1
80–84	-	-	-	-	4.8	23.2	55.4	274.8
≥85 years	-	-	-	-	3.1	22.8	39.4	310.2

Abbreviations: DME = diabetic macular edema, PDR = proliferative diabetic retinopathy.

## Data Availability

The data presented in this study are available in this article.

## Abbreviations

anti-VEGF	Vascular epithelial growth factor inhibitor
DME	Diabetic macular edema
ICD-10	International Classification of Diseases, revision 10
INDS	Institute of Health Data (France)
INSEE	National Institute of Statistics and Economic Studies (France)
nAMD	Neovascular age-related macular degeneration
PDR	Proliferative diabetic retinopathy
PRP	Pan-retinal photocoagulation
SNDS	National Health Insurance database (Système National des Données de Santé, France)
